# Long-Term Outcomes after Aortic Valve and Root Replacement in a Very High-Risk Population

**DOI:** 10.3390/jcdd9060197

**Published:** 2022-06-20

**Authors:** Jan Hlavicka, Kiril Antonov, Razan Salem, Florian Hecker, Spiros Marinos, Medhat Radwan, Fabian Emrich, Arnaud Van Linden, Anton Moritz, Thomas Walther, Tomas Holubec

**Affiliations:** 1 Department of Cardiovascular Surgery, University Hospital Frankfurt and Goethe University Frankfurt, 60590 Frankfurt/Main, Germany; jan.hlavicka@kgu.de (J.H.); kiril.antonov@kgu.de (K.A.); razan.salem@kgu.de (R.S.); florian.hecker@kgu.de (F.H.); fabian.emrich@kgu.de (F.E.); arnaud.vanlinden@kgu.de (A.V.L.); moritzanton@web.de (A.M.); thomas.walther@kgu.de (T.W.); 2 Division of Thoracic and Cardiovascular Surgery, University Hospital Tübingen, 72076 Tübingen, Germany; spiros.marinos@med.uni-tuebingen.de (S.M.); medhat.radwan@med.uni-tuebingen.de (M.R.)

**Keywords:** aortic valve and root replacement, Bentall–De Bono operation, high-risk population

## Abstract

**Background**: Aortic valve and root replacement (AVRR) is a standardised procedure to treat patients with aortic valve and root disease. In centres with a well-established aortic valve and root repair program (valve repairs and Ross operations), only patients with very complex conditions receive AVRR; this procedure uses a mechanical or biological composite valve graft (modified Bentall–de Bono procedure). The aim of the study was to evaluate the short- and long-term results after AVRR in a high-risk population with complex pathologies. **Methods**: Between 2005 and 2018, a total of 273 consecutive patients (mean age 64 ± 12.8 years; 23% female) received AVRR. The indication for surgery was an acute type A aortic dissection in 18%, infective endocarditis in 36% and other pathologies in 46% patients; 39% were redo procedures. The median EuroSCORE II was 11.65% (range 1.48–95.63%). Concomitant surgery was required in 157 patients (58%). **Results**: The follow-up extended to 5.2 years (range 0.1–15 years) and it was complete in 96% of the patients. The 30-day mortality was 17%. The overall estimated survival at 5 and 10 years was 65% ± 3% and 49% ± 4%, respectively. Univariate and multivariate logistic regression analyses revealed the following risk factors for survival: perioperative neurological dysfunction (OR 5.45), peripheral artery disease (OR 4.4) and re-exploration for bleeding (OR 3.37). **Conclusions**: AVRR can be performed with acceptable short- and long-term results in a sick patient population. The Bentall–De Bono procedure may be determined to be suitable for only elderly or high-risk patients. Any other patients should receive an AV repair or the Ross procedure in well-established centres.

## 1. Introduction

Aortic valve and root replacement (AVRR) using a composite valve graft (CVG) as described by Bentall and De Bono (BD) in 1968 [1] has become (through various modifications) the gold standard in the treatment of aortic root pathologies with or without aortic valve (AV) disease [2,3]. Simultaneously, the Ross procedure was introduced, particularly for younger patients with similar pathologies [4]; its use has increased, based on strong evidence [5,6]. Two decades later, valve-sparing operations such as aortic root remodelling (the Yacoub procedure) [7] and AV reimplantation (the David procedure) [8] were introduced. They allow for the preservation of native AV cusps and have excellent short- and long-term results [9,10,11]. Due to above mentioned reasons, these procedures have become the standard at many centres, especially in elective and low-risk patients [12]. Consequently, patients receiving AVRR carry a higher risk profile. The aim of our study was to assess the short- and long-term outcomes of AVRR using a CVG (modified BD procedure) under these conditions.

## 2. Materials and Methods

### 2.1. Ethics Statement

The study was conducted in accordance with the Declaration of Helsinki (as revised in 2013). It was approved by the local Ethics Committee of the University Hospital, Frankfurt, Germany (reference number: 154/18; first approval date: 19 July 2018 and most recent approval date: 10 June 2020). Written informed consent was obtained from each patient.

### 2.2. Study Design and Patient Selection

**Figure 1:**
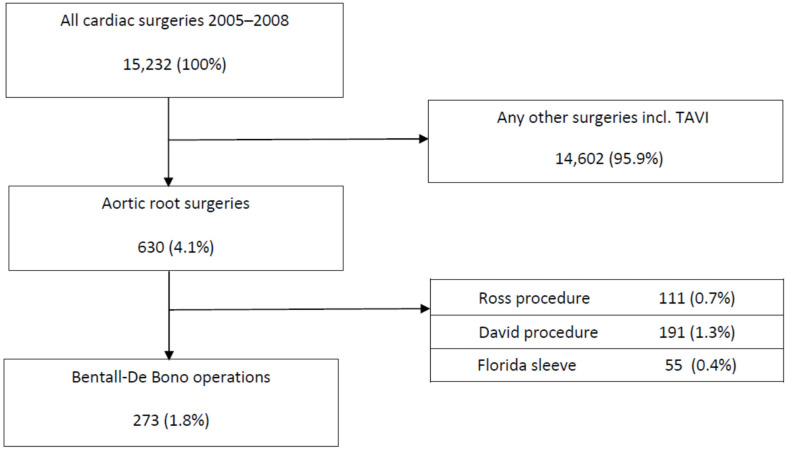
CONSORT-type diagram of patients with aortic valve and aortic pathology undergoing a Bentall–De Bono procedure. TAVI: transcatheter aortic valve implantation.

This single-centre study included a retrospective review and analysis of the pre-, peri- and postoperative records and protocols of patients who underwent AVRR with a composite graft according to the BD technique at the University Hospital, Frankfurt/Main, Germany, between 2005 and 2018. In total, 15,232 patients were operated on in this period of time at our department. Of them, 630 underwent an aortic root procedure. In 273 consecutive patients with a mean age of 64 ± 12.8 years (22.7% females), a BD procedure using a CVG (mechanical or biological) was performed. All consecutive patients were included in the study. Although aortic disease and a few forms of AV dysfunction were present in all patients (minor to severe cases), we identified three aortic root pathologies as a main indication for the BD operation at our department: acute type A aortic dissection, infectious endocarditis and a valve dysfunction (including valve stenosis, insufficiency or a combination of both) (Figure 1). The primary endpoint of the study was all-cause mortality. The secondary endpoint was freedom from reoperation.

### 2.3. Patient Management

All patients received a routine preoperative transthoracic echocardiography (TTE) as well as a coronary angiography and/or a computed tomography angiography. An intraoperative transoesophageal echocardiography was performed for all patients to facilitate the surgical procedure and to assess the implanted valve prosthesis and left ventricular function during weaning from a cardiopulmonary bypass. The postoperative anticoagulation regimen included initial subcutaneous low-molecular weight heparin for the first days in parallel with oral antiplatelet therapy with aspirin. Oral anticoagulation with coumadin was initiated only in patients with a mechanical CVG or a repeat postoperative atrial fibrillation. All patients received a TTE at discharge. All patients were prospectively followed by means of regularly mailed questionnaires or phone interviews and/or by a clinical assessment and TTE at our outpatient clinic. For patients not seen personally, we retrieved the clinical assessment and echocardiography reports from the attending cardiologist.

A follow-up was completed in 96% (10 patients were lost to the follow-up) with a median of 5.2 years (range 0.1–15 years), the total follow-up was 1725 patient/years. All patients who received an AV-sparing root replacement, isolated aortic valve replacement or AVRR with a homograft were excluded from this study.

### 2.4. Surgical Technique

The majority of patients were operated on through a median sternotomy and the minority via a partial upper sternotomy, as previously reported [13]. Arterial cannulation was performed either through the right axillary artery or the proximal aortic arch. A left ventricular vent was placed through the heart apex. After clamping the ascending aorta, a longitudinal aortotomy was performed with an extension into the non-coronary sinus of Valsalva. A cold blood cardioplegic solution (Calafiore) was directly infused into the coronary ostia to protect the heart. After resecting the aorta, the sizing of the aortic annulus was performed and the CVG was implanted with a series of interrupted pledget-everted 2-0 sutures with Ticron. The ‘button technique’ of the BD procedure was used in all patients. Finally, the anastomosis of the distal ascending aorta to the CVG with continuous 3-0 or 4-0 Prolene sutures was accomplished. In cases of additional aortic arch surgery, a hypothermic circulatory arrest with a selective bilateral antegrade cerebral perfusion was established via the right axillary artery and left common carotid artery.

### 2.5. Statistical Analysis

All data available were retrospectively collected and entered in a Microsoft Office Excel^®^ (Microsoft Corp, Redmond, WA, USA) spreadsheet. The continuous and discrete variables were reported as a mean ± standard deviation or a median and range for the data not normally distributed. The Kolmogorov–Smirnov test was used to assess the normality of the data. The categorical and ordinal variables were reported using the number and percentage of the observations. The continuous and discrete variables were compared using a Kruskal–Wallis or Mann–Whitney test where appropriate. The categorical and ordinal variables were compared using a Pearson chi-squared test or Fischer’s exact test where appropriate. Univariate and multivariate logistic regression analyses of the predictors for mortality were performed to evaluate the association between the independent risk factors, comorbidities and mortality. A multivariate Cox regression identified the independent risk factors of long-term mortality after a Bentall procedure. The probability of survival and freedom from the event was calculated according to the Kaplan–Meier method. Survival and freedom from the event curves were compared by a log-rank test. A two-sided *p*-value less than 0.05 was defined as indicating a statistical significance. The statistical analysis was performed using the IBM^®^ SPSS^®^ Statistics software program (version 25.0.0.0 for MS Windows, IBM Corporation, Armonk, NY, USA).

## 3. Results

### 3.1. Patient Characteristics

Between May 2005 and December 2018, a total of 273 consecutive patients underwent AVRR using either a mechanical or biological CVG (a modified BD) at our institution. Three AV and aortic root pathologies as main indications for the BD operation were identified: aortic dissection (18%), endocarditis (36%) and other pathologies (46%). Other pathologies were AV stenosis, insufficiency or a combination of both and aortic root dilatation. A total of 48% of the patients were operated on as urgent or an emergency. The baseline characteristics of all patients are listed in Table 1.

Of the patients, 78% were male. The patients in the endocarditis group were more often associated with a previous cardiac surgery. Preoperative echocardiography showed a significantly larger diameter of the ascending aorta in patients operated on due to dissection. The calculated median risk of the overall cohort according to EuroSCORE II was 11.65% (range 1.48–95.63 and IQR 18.28%).

### 3.2. Operative Data

The operative data of all patients are presented in Table 2.

Patients with an aortic dissection received a mechanical CVG more frequently than the other groups (*p* < 0.001). In addition, they most frequently received their operation as an emergency procedure. Concomitant mitral and tricuspid valve surgery was performed in patients suffering from endocarditis or in the group of patients with other pathologies. A total of 32 patients received concomitant mitral valve surgery and 26 patients had a tricuspid valve repair.

### 3.3. Early Postoperative Outcomes

The early postoperative data are presented in Table 3.

The overall 30-day mortality was 17.2% (47 of 273 patients). The mortality rates significantly differed between the groups and were the highest in patients with endocarditis (*p* = 0.004; Table 3). The causes of death were multiorgan failure in 13 (4.8%), low cardiac output syndrome in 12 (4.4%), severe bleeding in 11 (4%), sepsis in 7 (2.6%) and a cerebrovascular event in 4 (1.5%). Patients in the group of other pathologies suffered fewer strokes (6% vs. 6% vs. 0.8%, respectively; *p* = 0.003). Among the patients operated on due to dissection, atrial fibrillation occurred less frequently postoperatively compared with the two other groups (2% vs. 14% vs. 19%, respectively; *p* = 0.002).

### 3.4. Late Postoperative Outcomes

**Figure 2:**
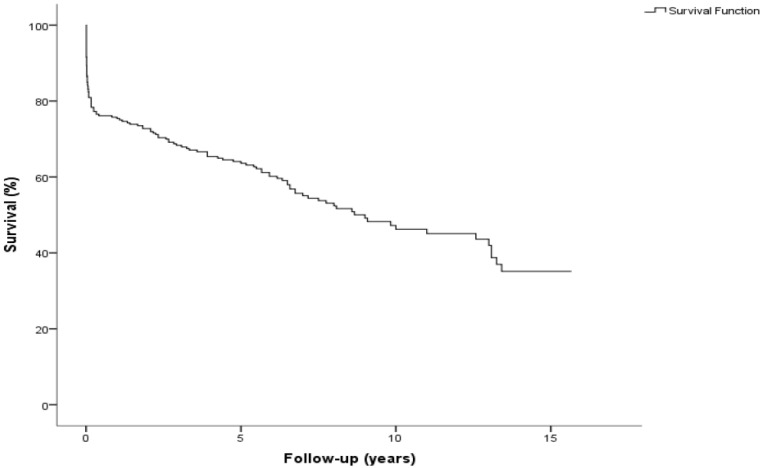
Kaplan-Meier curve showing overall survival after Bentall-De Bono operation. Pts, patients.

**Figure 3:**
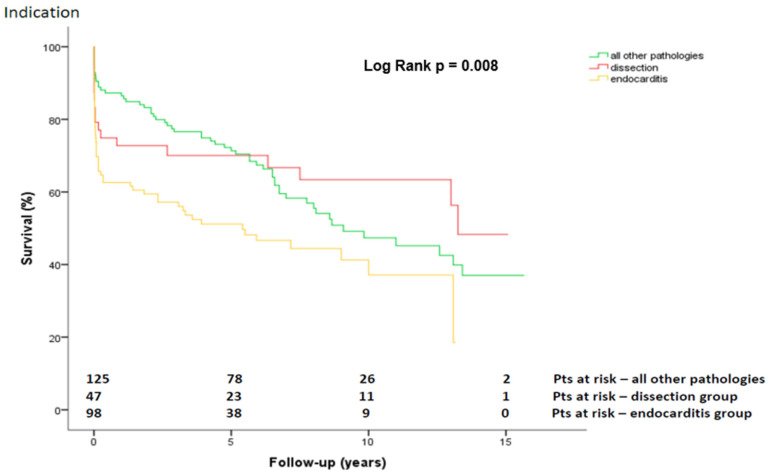
Kaplan-Meier curve showing survival after Bentall-De Bono operation in dissection versus endocarditis versus other pathologies group. Pts, patients.

**Figure 4:**
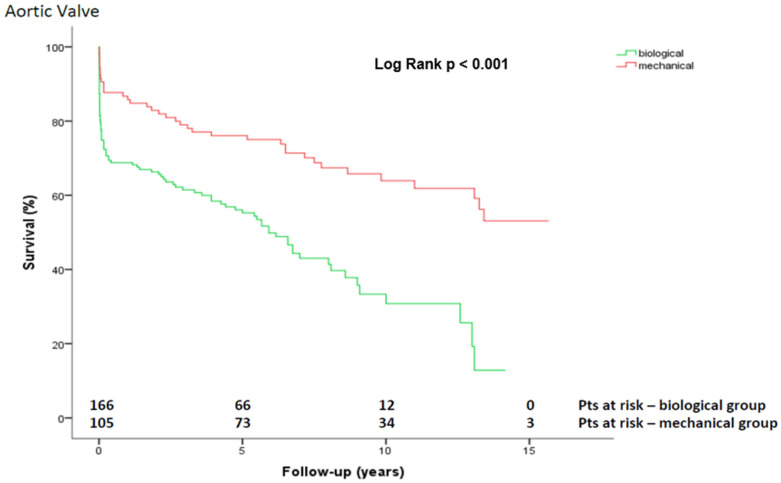
Kaplan-Meier curve showing survival after Bentall-De Bono operation using a biological versus mechanical composite valve graft. Pts, patients.

During follow-up, 130 (47.6%) patients died. The overall survival was 75.4 ± 2.6%, 63.6 ± 3.0%, and 46.2 ± 3.7% at 1, 5 and 10 years, respectively (Figure 2). The Kaplan–Meier survival estimation for the overall cohort was 8.6 ± 0.4 years (CI 7.7–9.4 years). After dividing the overall cohort into three groups based on underlying pathology, the survival at 1, 5, 10, and 15 years was 72.7 ± 6.5%, 70.1 ± 6.8%, 63.4 ± 7.6%, and 48.3 ± 11.0% (aortic dissection) vs. 62.6 ± 4.9%, 51.2 ± 5%, 37.1 ± 6.6%, and 18.6 ± 13.5% (endocarditis) vs. 86.5 ± 3.1%, 71.3 ± 4.1%, 47.3 ± 5.4%, and 37.0 ± 6.3% (other pathologies), respectively (*p* = 0.008; Figure 3). After dividing the overall cohort into mechanical and biological CVGs (age: 54.8 ± 12 vs. 69.9 ± 9.3 years; *p* < 0.001), = survival at 1, 5, 10, and 15 years was 85.8 ± 3.4%, 76.1 ± 4.2%, 63.9 ± 5.2%, and 53.1 ± 6.6% in the mechanical group vs. 68.8 ± 3.6%, 55.2 ± 4.0%, 30.8 ± 5.4%, and 12.8 ± 7.2% in the biological group, respectively (*p* < 0.001; Figure 4).

Only 12% of the patients suffered from complications during the follow-up period. Thromboembolism occurred in 6%, endocarditis in 6% and AV dysfunction in 3%, respectively. Univariate and multivariate analyses revealed the following risk factors for mortality after the BD procedure: postoperative neurological dysfunction (OR 5.45; 95% CI 1.172–25.416; *p* = 0.031), peripheral artery disease (OR 4.4; 95% CI 1.214–16.000; *p* = 0.024), in-hospital re-exploration due to bleeding (OR 3.37; 95% CI 1.751–6.429, *p* < 0.001), postoperative renal replacement therapy (OR 3.09; 95% CI 1.705–5.616; *p* < 0.001) and diabetes (OR 2.65; 95% CI 1.309–5.368; *p* = 0.007).

**Figure 5:**
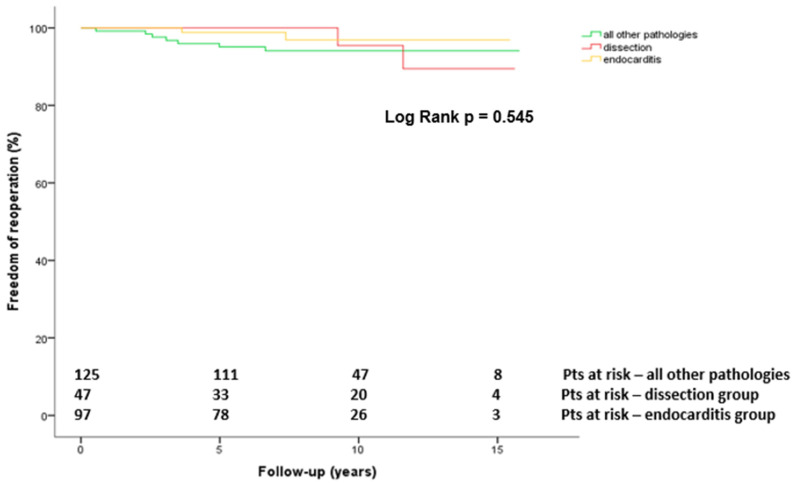
Kaplan-Meier estimated freedom of reoperation after Bentall-De Bono operation in dissection versus endocarditis versus other pathologies group. Pts, patients.

Only 11 (8.2%) of the surviving patients required a reoperation during the follow-up period. The most common indication for a reoperation was endocarditis (8/11), followed by a valve dysfunction (2/11) and a pseudoaneurysm in the ascending aorta (1/11). In the overall population, the 5- and 10-year freedom from reoperation of the AV and/or aortic root was 97.2 ± 1.0% and 95.2 ± 1.5%, respectively. After dividing the overall cohort into 3 groups based on the underlying pathology, the freedom from reoperation at 10 years was 95.2 ± 4.6% in the dissection group, 96.8 ± 2.3% in the endocarditis group and 93.9 ± 2.2% in the all other pathologies group (*p* = 0.545; Figure 5). The overall freedom from endocarditis at 10 years was 97.8 ± 1%. A statistically significant difference between the groups could not be detected (*p* = 0.689).

## 4. Discussion

In the present study, we aimed to assess the early and long-term outcomes after AVRR using a BD operation in high-risk patients with a particular regard for survival and freedom from reoperation. We analysed three different groups of pathologies and included a total number of 273 patients. The 30-day mortality in our study was 17.2%, which was higher than that reported by Mookhoek et al. in their meta-analysis (5.6%) [14]. The high mortality could most likely be explained by the high-risk profile of our patients. As a well-established centre for complex aortic pathologies as well as AV and root repair techniques, our department generally admits severely diseased patients; the mean age of all patients in our study was 64 years, 44% requested a concomitant valve or coronary procedure and 41.4% of the surgeries were reoperations, which dramatically increased the operative risk. Almost half of the operations (48%) were performed with an emergency or urgent label. Compared with the current results, the David procedure, performed among considerably younger and lower risk patients in our department, was burdened with only 1.4% 30-day mortality [15]. Similarly, a low early mortality of 1% after a Ross procedure in 2444 considerably younger and lower risk patients of the Ross registry that included over 200 patients from our department was recently published [5]. The median of the expected 30-day mortality of the overall cohort according to EuroSCORE II was 11.65% (range 1.48–95.63, IQR 18.28). Compared with the observed 30-day mortality of 17%, we reported relatively good results considering that EuroSCORE II can underestimate the operative risk, especially in high-risk patients, as Hogervorst et al. recently published [16].

In our experience, the mean survival was 8.6 ± 0.4 years (CI 7.7–9.4 years) after this procedure and the overall survival rate at 10 years was 46.2 ± 3.7%. Given the complexity of our cases and interventions, we believe that the overall survival in our analysis was satisfactory and in line with other reports [17,18]. Other studies have presented higher survival rates than this study; however, their patients were younger [14,19]. The higher survival rate in the patients with an aortic dissection compared with those with endocarditis or other aortic diseases (*p* < 0.01) was surprising and contradicted other studies [20]. The higher survival rates of the dissection group of 63.4 ± 7.6% and 48.3 ± 11.0% at 5 and 10 years, respectively, were probably based on their lower age and fewer comorbidities (Table 1) and, therefore, fewer in-hospital complications (Table 3. In our series, the mean age of the dissection group was approximately 10 years lower than in the two other groups. The dissection group was also the most homogenous and had no need of concomitant valve surgery. The patients in the endocarditis group had the worst survival rate of all groups; this was based on their high-risk profile, which included a high rate of previous cardiac surgery (80%), heart failure upon admission (51%) and preoperative strokes (34%). Additionally, a high percentage of intravenous addicts associated with a low level of compliance might have led to such a high mortality.

The observed and significantly lower survival of patients who received a biological CVG (*p* < 0.001) could be a result of the mean age of 69.9 ± 9.3 (range 36–89) of this subgroup, and the clinical practice of implanting a biological prosthesis in elderly patients and patients with relevant life-shortening comorbidities. The average life expectancy at birth in Germany between 1940 and 1950 was 61.6 to 63.7 years, respectively, and in 2019 was 80.19 years (77.93 for men and 82.58 for women). Therefore, the 15-year mortality of more than 80% of patients after cardiac surgery in the population aged almost 70 years is to be expected and cannot automatically be compared with the mechanical group; it should be statistically interpreted very cautiously. An increased number of cardiac decompensations were preoperatively noted; previous heart surgeries (including redo BD procedures), an increased burden of coronary heart disease, and COPD might also have had a negative impact on the survival of the patients with a biological CVG. Amongst the other factors, we identified biological valve grafts as an independent predictor of late mortality (OR 2.481; 95% CI 1.675–3.674; *p* < 0.001). Our results were in line with those of Pantaleo et al., who also recommend the use of mechanical valve grafts as the first choice of treatment if no contraindications are present [21].

Etz et al. identified independent risk factors for death after a BD procedure such as smoking, concomitant coronary surgery, a reoperation, the emergency nature of the surgery and the presence of an intracardiac clot. Their combination could substantially increase the in-hospital and late mortality [12]. On the contrary, we identified neurological dysfunction, peripheral artery disease, in-hospital re-exploration, postoperative renal replacement and diabetes as predictors for mortality. Only concomitant surgery due to coronary artery disease was identified as a relevant factor that increased the survival rate.

It is widely debated whether Marfan syndrome is associated with a decreased long-term survival or not [22]. We could not prove that Marfan syndrome was an independent risk factor for early or late mortality. All patients with this confirmed pathology survived to the 15-year follow-up in our study. Nevertheless, the patients with Marfan syndrome were considerably younger at the time of surgery. Improvements in operating techniques and postsurgical management may contribute to the low rate of their postoperative complications.

The reoperation rate in our study was low and in line with other published studies [19,23]. Similar to Pantaleo et al. and Prifti et al., we were able to identify infective endocarditis as the most serious cause of AV and/or aortic root reoperation with 8 of the 11 patients undergoing a reoperation due to endocarditis during the follow-up period [21,24].

The long-term complication rate was low with only 12% of surviving patients experiencing endocarditis, a thromboembolism or structural or non-structural AV prosthesis deterioration. Freedom rates from thromboembolisms, bleeding, endocarditis and valve-related complications were very satisfactory and also in line with other published studies [17,19].

### Study Limitations and Strengths

There were several limitations to this study. This was a retrospective, single-centre, non-randomised, observational study and all inherent disadvantages applied. The numbers of patients in the different groups were limited and the follow-up period was only long-term. Although the inclusion criteria were applied, the subjective decision-making of the involved surgeons based on their personal experience could not be excluded. Concomitant surgery on the aortic arch in the dissection group, associated with a longer cross-clamp time and hypothermic arrest, may have influenced the surgeon to perform a simplified AVRR rather than a more complex aortic valve reimplantation (the David procedure). Our patients were mostly high-risk, which negatively influenced the results and were not comparable with studies that only involved elective surgeries.

To the best of our knowledge, this study provides the first assessment of the long-term results of a BD operation in this specific high-risk population. The derived information could help surgeons with their decision-making process.

## 5. Conclusions

In patients with a very high-risk profile, AVRR using a CVG can be performed with an acceptable mortality, a low morbidity and a low rate of reoperation. At present, the Bentall–De Bono procedure should be determined only for elderly or high-risk patients. Other patients should receive an AV repair or a Ross procedure in well-established centres. The Bentall–De Bono procedure performed on this group of patients was associated with a higher risk than the EuroSCORE II could estimate. Further studies with a larger number of patients (multicentre) and a longer follow-up are warranted.

## Figures and Tables

**Table 1:** Baseline characteristics of the overall patient cohort divided according to the three main pathologies.

Variable	Overall (*n* = 273)	Dissection (*n* = 48)	Endocarditis (*n* = 99)	All Other Pathologies (*n* = 126)	*p*-Value
Mean age ± SD (years)	64 ± 12.8	54.8 ± 14.5	65.8 ± 12.8	66.2 ± 10.4	0.10
Female	62 (22.7%)	12 (25%)	22 (22%)	28 (22%)	0.92
NYHA ˃ II	171 (62.6%)	26 (54%)	73 (73%)	72 (57%)	<0.001
Atrial fibrillation	59 (21.6%)	0	30 (30%)	29 (23%)	<0.001
Pacemaker rhythm	19 (7%)	2 (4%)	6 (6%)	11 (9%)	0.006
Previous cardiac surgery	113 (41.4%)	2 (4%)	79 (80%)	32 (25%)	<0.001
CAD	101 (36.9%)	9 (19%)	41 (41%)	51 (40%)	0.017
Hypertension	250 (91.6%)	39 (81%)	88 (89%)	123 (98%)	0.001
Pulmonary hypertension	105 (38.5%)	3 (6%)	48 (48%)	54 (43%)	<0.001
Heart failure upon admission	98 (35.9%)	12 (25%)	50 (51%)	36 (29%)	0.001
PAD	16 (5.8%)	3 (6%)	8 (8%)	5 (4%)	0.42
Diabetes	43 (15.7%)	6 (13%)	21 (21%)	16 (13%)	0.174
COPD	90(32.9%)	4 (8%)	38 (38%)	48 (38%)	<0.001
Preoperative stroke	50 (18.3%)	5 (10%)	34 (34%)	11 (9%)	<0.001
Renal insufficiency	97 (35.5%)	15 (31%)	43 (43%)	39 (31%)	0.12
Bicuspid AV	70 (25.6%)	11 (23%)	9 (9%)	50 (40%)	<0.001
EF (%)	56.428	58.41	55.894	56.53	0.96
AV gradient mean (mmHg)	24.49	17.15	20.06	27.87	0.90
Ascending aortic diameter (mm)	44.09	56.58	35.56	47.00	<0.001
AR ˃ moderate	91 (33.3%)	21 (42%)	31 (31%)	39 (28%)	0.002

AR: aortic regurgitation; AV: aortic valve; CAD: coronary artery disease; COPD: chronic obstructive pulmonary disease; EF: ejection fraction; PAD: peripheral artery disease; SD: standard deviation.

**Table 2:** Operative data.

Variable	Overall (*n* = 273)	Dissection Group (*n* = 48)	Endocarditis Group (*n* = 99)	All Other Pathologies (*n* = 126)	*p*-Value
Timing of operation					<0.001
Emergency	62 (22.7%)	44 (92%)	15 (15%)	3 (2%)	
Urgent	69 (25.3%)	2 (4%)	45 (46%)	22 (18%)	
Elective	142 (52%)	2 (4%)	39 (39%)	101 (80%)	
AV prosthesis					<0.001
Biological	167 (61.2%)	14 (29%)	78 (79%)	75 (60%)	
Mechanical	106 (38.8%)	34 (71%)	21 (21%)	51 (40%)	
Conduit diameter, mean (mm)	25.1	25.5	24.6	25.4	0.05
Concomitant surgery					<0.001
CABG	62 (22.7%)	10 (21%)	14 (14%)	38 (30%)	
MV replacement	6 (2.2%)	0	3 (3%)	3 (2%)	
MV repair	26 (9.5%)	0	12 (12%)	14 (11%)	
TV repair	26 (9.5%)	0	8 (8%)	18 (14%)	
Minimally invasive	48 (17.6%)	2 (4%)	10 (10%)	36 (29%)	
CPB time, median (range), minutes	193 (477–60)	213 (477–106)	211 (431–107)	173 (439–60)	0.58
Cross-clamp time, median (range), minutes	131 (374–47)	137 (374–69)	140 (239–71)	122 (253–47)	0.72

AV: aortic valve; CABG: coronary artery bypass grafting; CPB: cardiopulmonary bypass; MV: mitral valve; TV: tricuspid valve.

**Table 3:** Early postoperative data.

Variable	Overall (*n* = 273)	Dissection (*n* = 48)	Endocarditis (*n* = 99)	All Other Pathologies (*n* = 126)	*p*-Value
Re-exploration for bleeding	55 (20.1%)	9 (19%)	22 (22%)	24 (19%)	0.81
Stroke	13 (4.7%)	3 (6%)	6 (6%)	1 (0.8%)	0.003
Renal failure (dialysis)	68 (24.9%)	11 (23%)	39 (40%)	18 (14%)	< 0.001
Permanent pacemaker	24 (8.8%)	7 (15%)	11 (11%)	6 (5%)	0.07
Myocardial infarction	7 (2.6%)	1 (2%)	3 (3%)	3 (2%)	0.93
Pericardial effusion	59 (21.6%)	9 (19%)	27 (27%)	23 (18%)	0.81
Wound healing disorder	20 (7.3%)	2 (4%)	9 (9%)	9 (7%)	0.62
New onset AF	39 (14.3%)	1 (2%)	14 (14%)	24 (19%)	
30-day mortality	47 (17.2%)	9 (19%)	26 (26%)	12 (10%)	0.004

AF: atrial fibrillation.

## Data Availability

The data that support the findings of this study are available on request from the corresponding author.
