# Correction: Hlavicka et al. Long-Term Outcomes after Aortic Valve and Root Replacement in a Very High-Risk Population. *J. Cardiovasc. Dev. Dis.* 2022, *9*, 197

**DOI:** 10.3390/jcdd10120500

**Published:** 2023-12-18

**Authors:** Jan Hlavicka, Kiril Antonov, Razan Salem, Florian Hecker, Spiros Marinos, Medhat Radwan, Fabian Emrich, Arnaud Van Linden, Anton Moritz, Thomas Walther, Tomas Holubec

**Affiliations:** 1 Department of Cardiovascular Surgery, University Hospital Frankfurt and Goethe University Frankfurt, 60590 Frankfurt/Main, Germany; jan.hlavicka@kgu.de (J.H.); kiril.antonov@kgu.de (K.A.); razan.salem@kgu.de (R.S.); florian.hecker@kgu.de (F.H.); fabian.emrich@kgu.de (F.E.); arnaud.vanlinden@kgu.de (A.V.L.); moritzanton@web.de (A.M.); thomas.walther@kgu.de (T.W.); 2 Division of Thoracic and Cardiovascular Surgery, University Hospital Tübingen, 72076 Tübingen, Germany; spiros.marinos@med.uni-tuebingen.de (S.M.); medhat.radwan@med.uni-tuebingen.de (M.R.)

The authors would like to make the following corrections to the published paper [1].

The author wishes to revise the caption of Figures 2–5 due to erratum. The Figures do not have to be changed; the spelling patterns of “patients”, “Kaplan–Meier”, “Bentall–DeBono” and “vs.” have been modified in Figures 2–5. The corrected captions of Figures 2–5 appear below. 

**Figure 2.** Kaplan-Meier curve showing overall survival after Bentall-De Bono operation. Pts, patients.

**Figure 3.** Kaplan-Meier curve showing survival after Bentall-De Bono operation in dissection versus endocarditis versus other pathologies group. Pts, patients.

**Figure 4.** Kaplan-Meier curve showing survival after Bentall-De Bono operation using a biological versus mechanical composite valve graft. Pts, patients.

**Figure 5.** Kaplan-Meier estimated freedom of reoperation after Bentall-De Bono operation in dissection versus endocarditis versus other pathologies group. Pts, patients.

There was an error in the original publication. After rechecking the text of our article, we identified a certain inaccuracy and accomplished some additional information. We noticed that the data we published are not correct. In the original version, we mentioned years 1, 5, and 10, but the data correspond to 5, 10, and 15 years. This meant that initially, data for year 1 was missing. Now, we wish to publish the data from years 1, 5, 10, and 15, adding the 1-year data. The data published now correspond with the commentary perfectly. 

A correction has been made to “3.4. Late Postoperative Outcomes”, in the first paragraph:

“During follow-up, 130 (47.6%) patients died. The overall survival was 75.4 ± 2.6%, 63.6 ± 3.0%, and 46.2 ± 3.7% at 1, 5 and 10 years, respectively (Figure 2). The Kaplan–Meier survival estimation for the overall cohort was 8.6 ± 0.4 years (CI 7.7–9.4 years). After dividing the overall cohort into three groups based on underlying pathology, the survival at 1, 5, 10, and 15 years was 72.7 ± 6.5%, 70.1 ± 6.8%, 63.4 ± 7.6%, and 48.3 ± 11.0% (aortic dissection) vs. 62.6 ± 4.9%, 51.2 ± 5%, 37.1 ± 6.6%, and 18.6 ± 13.5% (endocarditis) vs. 86.5 ± 3.1%, 71.3 ± 4.1%, 47.3 ± 5.4%, and 37.0 ± 6.3% (other pathologies), respectively (*p* = 0.008; Figure 3). After dividing the overall cohort into mechanical and biological CVGs (age: 54.8 ± 12 vs. 69.9 ± 9.3 years; *p* < 0.001), = survival at 1, 5, 10, and 15 years was 85.8 ± 3.4%, 76.1 ± 4.2%, 63.9 ± 5.2%, and 53.1 ± 6.6% in the mechanical group vs. 68.8 ± 3.6%, 55.2 ± 4.0%, 30.8 ± 5.4%, and 12.8 ± 7.2% in the biological group, respectively (*p* < 0.001; Figure 4).”

There was an error in the original publication. Based on a suggestion from the academic editor, the discussion took into count the average age of the operated patients and the life expectancy of the generation born between 1940 and 1950 in Germany. We find the discussion on this topic to be sufficient, so we added a sentence to clarify this.

A correction has been made to section “4. Discussion”, in the third paragraph:

“The observed and significantly lower survival of patients who received a biological CVG (*p* < 0.001) could be a result of the mean age of 69.9 ± 9.3 (range 36–89) of this subgroup, and the clinical practice of implanting a biological prosthesis in elderly patients and patients with relevant life-shortening comorbidities. The average life expectancy at birth in Germany between 1940 and 1950 was 61.6 to 63.7 years, respectively, and in 2019 was 80.19 years (77.93 for men and 82.58 for women). Therefore, the 15-year mortality of more than 80% of patients after cardiac surgery in the population aged almost 70 years is to be expected and cannot automatically be compared with the mechanical group; it should be statistically interpreted very cautiously. An increased number of cardiac decompensations were preoperatively noted; previous heart surgeries (including redo BD procedures), an increased burden of coronary heart disease, and COPD might also have had a negative impact on the survival of the patients with a biological CVG. Amongst the other factors, we identified biological valve grafts as an independent predictor of late mortality (OR 2.481; 95% CI 1.675–3.674; *p* < 0.001). Our results were in line with those of Pantaleo et al., who also recommend the use of mechanical valve grafts as the first choice of treatment if no contraindications are present [21].”

The authors apologize for any inconvenience caused, and state that the scientific conclusions are unaffected. This correction was approved by the academic editor. The original publication has also been updated.
